# The IgH locus 3′ cis-regulatory super-enhancer co-opts AID for allelic transvection

**DOI:** 10.18632/oncotarget.14585

**Published:** 2017-01-10

**Authors:** Sandrine Le Noir, Brice Laffleur, Claire Carrion, Armand Garot, Sandrine Lecardeur, Eric Pinaud, Yves Denizot, Jane Skok, Michel Cogné

**Affiliations:** ^1^ UMR 7276 CNRS and Université de Limoges: Contrôle de la Réponse Immune B et Lymphoprolifération, Limoges, France; ^2^ Department of Pathology, New York University School of Medicine, New York, NY, USA

**Keywords:** super-enhancer, alleles, transvection, nuclear positioning, gene regulation

## Abstract

Immunoglobulin heavy chain (*IgH*) alleles have ambivalent relationships: they feature both allelic exclusion, ensuring monoallelic expression of a single immunoglobulin (Ig) allele, and frequent inter-allelic class-switch recombination (CSR) reassembling genes from both alleles. The *IgH* locus 3′ regulatory region (3′RR) includes several transcriptional *cis*-enhancers promoting activation-induced cytidine deaminase (AID)-dependent somatic hypermutation (SHM) and CSR, and altogether behaves as a strong super-enhancer. It can also promote deregulated expression of translocated oncogenes during lymphomagenesis. Besides these rare, illegitimate and pathogenic interactions, we now show that under physiological conditions, the 3′RR super-enhancer supports not only legitimate *cis-*, but also *trans-*recruitment of AID, contributing to *IgH* inter-allelic proximity and enabling the super-enhancer on one allele to stimulate biallelic SHM and CSR. Such inter-allelic activating interactions define *transvection*, a phenomenon well-known in drosophila but rarely observed in mammalian cells, now appearing as a unique feature of the *IgH* 3′RR super-enhancer.

## INTRODUCTION

Interactions and co-localization of non-homologous genes are favored by super-enhancers, *i.e*. major *cis*-regulatory elements controlling key cell identity genes [1–4]. In B-cells, the specific and frequent co-localization of immunoglobulin heavy chain (*IgH*) genes, Ig κ light chain genes (*Igκ*) and J-chain genes (*IgJ*) has been reported at all stages of B-cell differentiation including plasma cells where high expression of these loci often involves the same transcription factories and a nuclear organization that may optimize the nuclear export of their transcripts and further translation [1, 3, 4]. Functional interactions between those co-localized non-homologous genes occur at the level of transcription, with the 3′Eκ enhancer positively influencing *IgH* transcription [3], but such effects were not evaluated between alleles of each locus.

Stimulatory interactions between homologous paired alleles, defining transvection, are well known in drosophila [5], but rarely observed in mammals except in pathologic or artificial settings. For example, transvection was documented between an *IgH*-translocated CCND1 gene and its non-translocated counterpart [6]. Manipulating the mouse genome also yielded transvection from a mutant paternal and expressed *Igfr2* allele which triggered expression of an otherwise silent maternal allele [7]. Inhibitory *trans*-regulation, supporting allelic exclusion, is by contrast frequent in mammalian physiology. Regarding *Ig* genes, ordered V(D)J recombination initiates allelic exclusion during early B cell development, when a functional rearrangement shuts off accessibility of the second allele, which correlates with modified epigenetic marks, interactions of *IgH/Igκ* loci, and changes in nuclear location [8, 9].

In mature activated B-cells, localization close to the nuclear periphery and co-localization with *Igκ* and *IgJ* genes preferentially mark the functionally rearranged *IgH* allele [3]. However, inter-allelic contacts are also repeatedly identified in 3C, 4C, and FISH-3D [3, 9, 10]. In parallel, it is noticeable that both transcription and AID-dependent *IgH* changes such as class switch recombination (CSR) and somatic hypermutation (SHM) usually affect both productive and non-productive alleles at similar levels [9, 11, 12].

In the *IgH* locus, the role of the 3′ regulatory region (3′RR) enhancers goes beyond transcription and also features recruitment of AID for CSR, SHM or deletional silencing of constant genes through locus suicide recombination [13–17]. The 3′RR super-enhancer features a high density of individual enhancers [15], eRNA transcription [13, 18], regulation by long non-coding RNA [4], active chromatin marks and the ability to promote higher-order chromosomal structures [19]. Its polymorphisms influence susceptibility to immune disorders, while it endows transgenes with B-cell specific expression and can also deregulate oncogenes [20–23]. As any *cis*-regulatory element, the 3′RR controls covalently linked genes within a legitimate territory bounded by insulators, and even the deregulation of oncogenes secondary to translocation falls into *bona fide* “*cis”* effects. It is by contrast unclear whether the gene co-localization observed in B-cells has functional implications in *trans*.

In this regard, while illegitimate off-target CSR-like junctions with non *Ig* genes remain exceptional during AID expression, legitimate junctions repairing CSR breaks are known to occur actively in both *cis* and *trans* [24–27]. Promiscuous *trans*-CSR junctions strongly suggest that *IgH* biallelic co-localization has functional implications at stages where proximity might secure legitimate synapsis between concomitantly generated biallelic DNA breaks. In order to explore whether these functional interactions precede *trans-*CSR, we thus searched for inter-allelic stimulatory interactions reminiscent to transvection, and witnessed pairing between homologous and legitimate AID target alleles. Since the 3′RR is a regulator of both long-range interactions and AID recruitment, we considered that inter-allelic proximity may yield legitimate effects in *trans* and in the absence of covalent linkage. Here we indeed demonstrate 3′RR-dependent IgH *trans*-interactions in B-cells with either additional copies of the 3′RR linked to transgenes or, by contrast, with homozygous or hemizygous loss of the 3′RR. The latter configuration also reveals the ability of the 3′RR super-enhancer to yield DNA accessibility and recruit AID not only in *cis* but also in *trans*.

## RESULTS

### 3′RR-dependent interactions of *IgH* transgenes with of endogenous *IgH* genes

**Figure 1:**
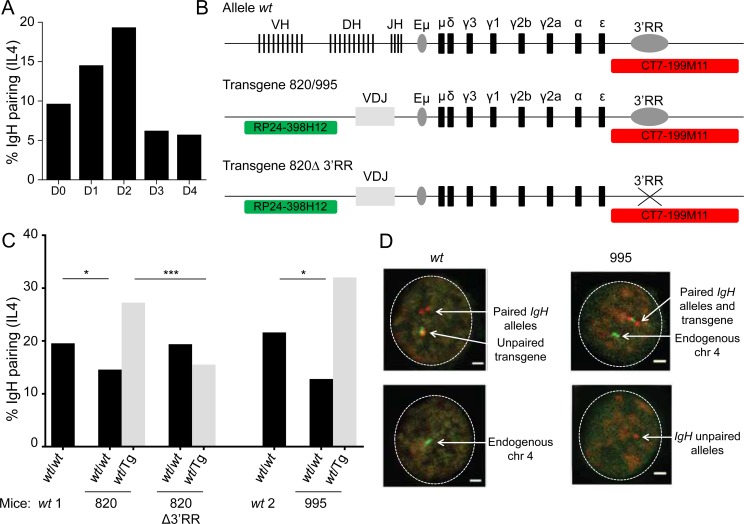
3′RR dependent *IgH* pairing in *wt* and transgenic B-cells **A**. Kinetic of *IgH* alleles pairing in *in vitro* activated *wt* B-cells. *Wt* B splenocytes were stimulated 4 days *in vitro* with anti-CD40 + IL4 and *IgH* localization was studied by 3D FISH. *IgH* pairing (distance ≤ 1 µm) was quantified in resting (day 0) and activated B cells (D1, D2, D3, D4). Bars represent % of pairing from 2 experiments with at least 585 cells analyzed. **B**. Schematic representation of alleles and probes used for 3D FISH experiments (not scaled). *Wt* alleles contain all V(D)J genes, constant genes and 3′RR, transgenes include a rearranged VDJ gene, constant genes and 3′RR (820 and 995 transgenes) or not (820Δ transgene). Probes are specific for *IgH* (CT7-199M11, red) or for transgene integration site (RP24-398H12, green, chromosome 4). **C**. *IgH* pairing in *in vitro* activated B lymphocytes. Cells were analyzed at day 2 and *IgH* pairing (distance ≤ 1 µm) between *wt* and *wt* alleles (black) or between *wt* and transgenic alleles (grey) were quantified by 3D FISH. In a first set of experiments *wt* cells (wt1) were compared to 820 and 820Δ transgenic cells and in a separated experiment *wt* cells (wt2) were compared to 995 transgenic cells. Bars represent % of pairing from 2 experiments, with at least 50 cells per group. DNA probes used were BAC CT7-199M11 for 3´ *IgH* and BAC RP24-221C18 close to the insertion site of the *IgH* transgene on chromosome 7 or BAC RP24-398H12, which is located adjacent to the insertion site of the *IgH* transgene on chromosome 4. Fisher exact test for significance. **D**. Representative nuclei from 3D FISH experiments on *in vitro* activated B-cells (day 2). Examples of pairing between endogenous *IgH* alleles in *wt* cells and between transgene and *IgH* alleles in 995 transgenic cells are shown. *IgH* loci are in red, transgene and endogenous chromosome 4 are in green. Scale bar: 1µm.

*IgH-Igκ* inter-chromosomic pairing has been shown to occur at all stages of B-cell differentiation (except pro-B), while *IgH-IgH* inter-allelic interactions were evaluated during V(D)J recombination in developing B-cells [10] and plasma cells [3]. We checked and validated that pairing of *IgH* alleles can also be detected by 3D FISH in mature resting and *in vitro* activated B-cells (Figure 1A).

To determine whether pairing of homologous *IgH* sequences depends upon the chromosomal context, we analyzed mice which carry an *IgH* transgene either as one copy on chromosome 7 (mouse line 820) or 3 copies on chromosome 4 (mouse line 995). This large *IgH* transgene included the *IgH* 3′RR (Figure 1B) [28]. In primary B-cells cultured *in vitro* (after 48h anti-CD40 + IL-4 stimulation of splenic cells from the 820 strain), we observed frequent pairing of the endogenous *IgH* locus with the *IgH* transgene. Pairing with the transgene reached a higher level (because there are two copies of the endogenous alleles with which it can pair) and competed with pairing of endogenous *IgH* alleles (Figure 1C and 1D). Similar results were obtained with cells from the second (995) transgenic mouse strain (Figure 1C and 1D). Together these experiments demonstrate that homologous *IgH* alleles or transgenes pair independent of chromosomal context.

### The 3′ enhancer is required for pairing of an *IgH* transgene with the endogenous locus

We next examined splenic B cells from 820Δ mice, which bear the 820 transgene after *cre*-mediated deletion of the entire 3′RR [28]. Consistent with previous analyses, we observed no cell surface expression of Ig encoded by the rearranged transgene in activated splenic B cells under switching conditions (Figure S1A). Further, the 820Δ cells expressed 3-fold less transgenic germline transcripts compared to 820 cells (Figure S1B). Importantly by comparison to the parental 820 strain cells, pairing of the mutant transgene with endogenous *IgH* alleles was clearly reduced, while pairing between both endogenous *IgH* alleles was restored to normal levels (Figure 1C). Thus, the ability of an *IgH* transgene to pair with the homologous endogenous locus strongly depends on its inclusion of the 3´RR.

### 3′RR-dependent nuclear positioning of endogenous *IgH* loci

**Figure 2:**
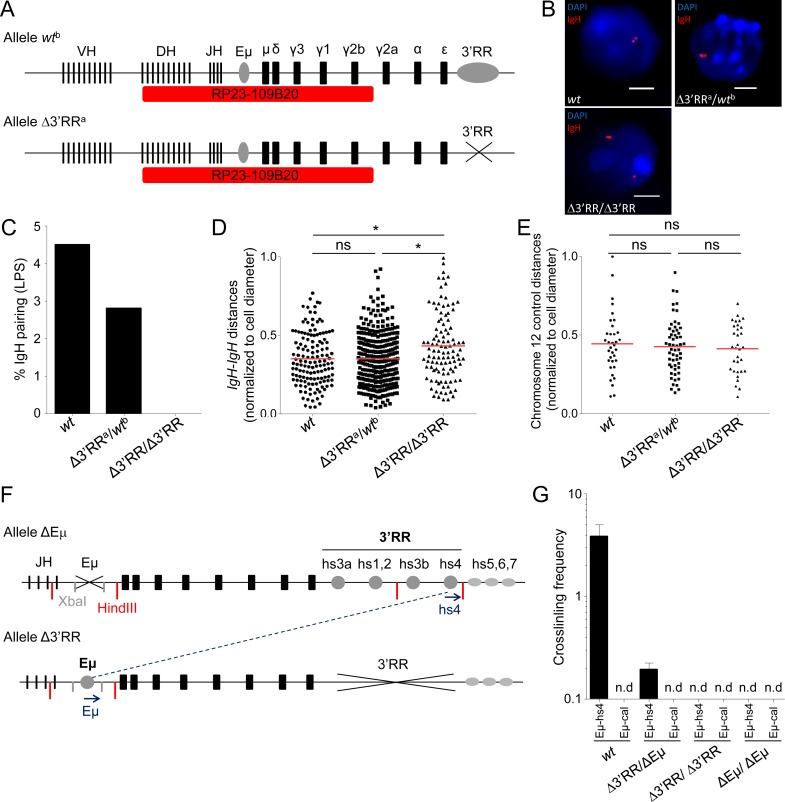
3′RR-dependent *IgH* inter-allelic interactions in *wt*, 3′RR hemizygous and 3′RR-deficient B cells **A**. Schematic representation of *IgH* alleles and probe (RP23-109B20, red) used for 3D FISH experiments (not scaled). *Wt*^b^ allele includes the 3′RR, contrary to the Δ3′RR^a^ allele. **B**. Representative nuclei from 3D FISH experiments on *in vitro* LPS activated B-cells (day 3). Examples of pairing observed in *wt* and Δ3′RR^a^/*wt*^b^ cells, Δ3′RR/Δ3′RR failed to pair their *IgH* alleles. DAPI is in blue, *IgH* loci are in red. Scale bars: 4µm. **C**. *IgH* pairing in *in vitro* LPS activated B lymphocytes. Cells were analyzed at day 3 and *IgH* pairing (distance ≤ 1 µm) between alleles was quantified by 3D FISH. Bars represent % of pairing from at least 2 experiments, corresponding to 138 (*wt*), 107 (Δ3′RR) and 324 (Δ3′RR^a^/*wt*^b^) cells analyzed. **D.** Distribution of IgH inter-allelic distance in activated B-cells (with normalization according to cell diameter) from *wt*, Δ3′RR and Δ3′RR^a^/*wt*^b^ cells are shown (from the same cells as in 2C). Mean is shown and KS test for significance. **E**. Distribution of inter-allelic distance (with normalization according to cell diameter) analyzed for a non-*IgH* chromosome 12 locus (probe RP23-436M24) in activated B-cells from *wt* (*n* = 35), Δ3′RR (*n* = 32) and Δ3′RR/*wt* (*n* = 56). (> 2 experiments with > 3 mice per group). Mean is shown and KS test for significance. **F**. Schematic representation of *IgH* ΔEµ and Δ3′RR alleles used for 3C experiments. Primers (blue arrows), XbaI (grey) and HindII (red) restriction sites are shown (not to scale). **G**. Relative inter-allelic interactions between *IgH* alleles determined by 3C experiments. Eµ-hs4 or Eµ-*calreticulin* (Eµ-*cal*) 3C crosslinking frequencies for *wt*, Δ3′RR/Δ3′RR, ΔEµ/ΔEµ and double heterozygous Δ3′RR/ΔEµ mice were determined from *in vitro* LPS activated B-cells (2 experiments with ≥ 3 mice per group). Mean is shown +/- s.e.m. (n.d., non-detected).

To evaluate whether inter-allelic pairing of endogenous *IgH* loci also relied on the 3′RR, inter-*IgH* distance was compared by 3D-FISH in *wt*, Δ3′RR^a^/*wt*^b^ and homozygous Δ3′RR^a^/Δ3′RR^a^ *in vitro* activated B-cells (72h with LPS) (Figure 2A and 2B). As for close inter-allelic interactions ≤ 1µm, none was detectable in Δ3′RR cells; they by contrast showed up in heterozygous cells, with a single 3′RR being sufficient to bring both *IgH* alleles into contact as in *wt* cells, albeit at a lower frequency (Figure 2C). In addition, the distribution of the *IgH*-*IgH* distance was clearly shifted toward increased distances for homozygous Δ3′RR B-cells compared to *wt* (*p* = 0.037) and Δ3′RR^a^/*wt*^b^ (*p* = 0.024) (Figure 2D). A control experiment with a distant non-*IgH* probe from the same chromosome 12 showed no variation in the inter-chromosomal distance, demonstrating that effects of the 3′RR deletion do not extend beyond the *IgH* locus (Figure 2E).

We also generated heterozygous Δ3′RR/ΔEµ mice lacking the 3′RR on one allele and Eµ on the other (Figure 2F), thereby suppressing known *cis*-interactions between these elements [19] in order to check whether inter-allelic interactions were still detectable in such conditions. 3C experiments in *in vitro* activated B-cells (72h with LPS) revealed persistent inter-allelic interactions, with Eµ from allele 1 contacting the 3′RR from allele 2. Parallel controls monitored the lack of any interaction with the unrelated *calreticulin* gene (as described in [19] (Figure 1G)).

Taken together these observations show a 3′RR-dependent inter-allelic interaction between *IgH* loci in mature B-cells, which might participate in the function of the super-enhancer.

### *Trans*-activation by the *wt*^b^ allele functional 3′RR during CSR

We explored if proximity and 3′RR *trans*-activation might support CSR on the second allele. Homozygous 3′RR deletions suppress CSR-accessibility of downstream switch (S) regions but have no effect on Sµ (and barely any on Sγ1) [17, 29]. This is partly rescued by heterozygosity, where *trans-*CSR is maintained and joins the accessible Sµ from the 3′RR-less allele to downstream S regions from the *wt* allele [24]. The *IgH*^a^ locus (from SV129 strain) drives expression of IgM^a^, IgG1^a^, IgG2a, IgE^a^ and IgA^a^ (for which some allotype-specific antisera are available) instead of IgM^b^, IgG1^b^, IgG2c, IgE^b^ and IgA^b^ for the *IgH*^b^ locus (from C57BL/6 strain). Whether using *cis-* or *trans-*CSR, heterozygous Δ3′RR^a^/*wt*^b^ mice should thus overwhelmingly secrete switched Ig from the accessible *IgH*^b^ constant genes, with the exception of IgG1 which is less affected by the 3′RR deletion in Δ3′RR^a^/Δ3′RR^a^ homozygous mice [17]. We analyzed whether in heterozygous settings, the 3′RR-proficient *IgH* ^b^ allele can rescue some usage of the 3′RR-dependent C genes (that are not accessible) from the 3′RR-deleted *IgH* ^a^ allele.

**Figure 3:**
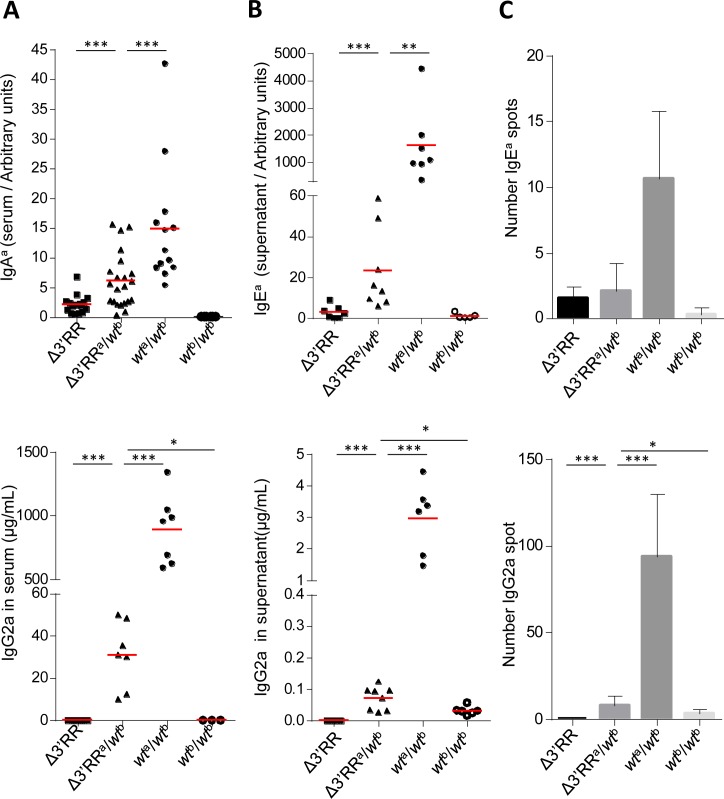
The 3′RR partially rescues CSR in *trans* on a 3′RR-*cis*-deficient *IgH* allele **A**. *In vivo IgH*^a^ allele CSR in Δ3′RR^a^/*wt*^b^ mice: IgG2a (top) and IgA^a^ (bottom) serum levels from homozygous Δ3′RR, Δ3′RR^a^/*wt*^b^, *wt*^a/b^ and *wt*^b/b^ mice. Each point represents one mouse, mean is shown (≥ 3 independent experiments). Mann-Whitney test for significance. **B**. *In vitro IgH*^a^ allele CSR in Δ3′RR^a^/*wt*^b^ cells: IgG2a (top) and IgE^a^ (bottom) levels in supernatant from Δ3′RR, Δ3′RR^a^/*wt*^b^*, wt*^a/b^ and *wt*^b/b^ stimulated B-cells. Each point represents one stimulation from one mouse, mean is shown (≥ 3 independent experiments). Mann-Whitney test for significance. **C**. ELISpot specific for IgG2a (top) and IgE^a^ (bottom) were performed on *in vitro* stimulated B-cells from Δ3′RR, Δ3′RR^a^/*wt*^b^*, wt*^a/b^ and *wt*^b/b^. Data shown are from 3 independent experiments with at least 2 mice. Mean +/- s.d. is shown. Mann-Whitney test for significance.

In sera, we observed that instead of following gene dosage, allotype-specific IgA^a^ and IgG2a levels (measuring Cγ^a^ and Cγ2a CSR on the Δ3′RR^a^ allele) significantly increased in heterozygous compared to homozygous Δ3′RR^a^ mice (Figure 3A). The same was true for *in vitro* stimulated B-cells both by evaluating secretion of the IgE^a^ and IgG2a allotypes in supernatants (Figure 3B) and by directly counting IgE^a^ and IgG2a switched plasma cells by ELISpot (Figure 3C). Rescued CSR-accessibility of 3′RR-deleted *IgH*^a^ C genes thus indicates inter-allelic 3′RR *trans*-activation.

### *Trans*-activation by the *wt*^b^ allele functional 3′RR during SHM

**Figure 4:**
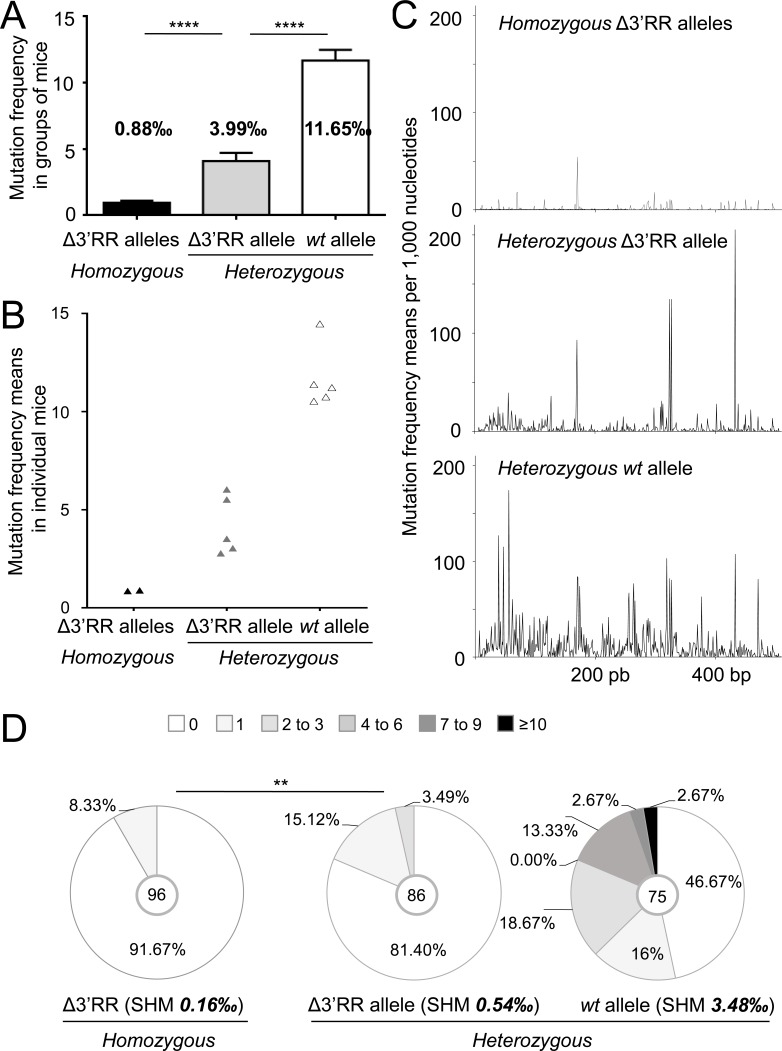
The 3′RR partially rescues SHM in *trans* on a 3′RR-*cis*-deficient *IgH* allele **A**. Mutation frequency in 3′J_H_4 intron from Peyer's patches (B220^+^/GL7^+^) in Δ3′RR/Δ3′RR mice and in Δ3′RR^a^ and *wt*^b^ alleles from Δ3′RR^a^/*wt*^b^ mice. Mean +/- s.d. is shown. Mann-Whitney test for significance. **B**. Mutation frequency as in (A), but with each point representing an individual mouse in order to evaluate the dispersion of the results when SHM is measured in groups of mice with homogeneous genotype. **C**. Graph of mutation frequencies along the sequenced J_H_4 flanking region in homozygous Δ3′RR mice (top) and in Δ3′RR^a^ (middle) and *wt*^b^ (bottom) alleles from Δ3′RR^a^/*wt*^b^ mice. **D**. Mutation frequencies in 3′J_H_4 intron in Peyer's patches B220^+^/GL7^+^/IgM^+^ cells from homozygous Δ3′RR and Δ3′RR^a^/*wt*^b^ heterozygous mice. Data were obtained from at least 3 mice per group and clonally related sequences were counted only once. Khi2 test for significance.

Potential *trans*-complementation of a SHM defect in Peyer's patches GC B-cells was checked downstream of rearranged J_H_4 segments (through either cloning/high-fidelity sequencing or high throughput next generation sequencing (NGS)). As previously documented [16], *IgH* SHM was deficient upon Δ3′RR homozygosity (0.89‰ bp in *IgH vs* 16.9‰ for *Igκ*) (Table 1, Figure 4A and 4B). In Δ3′RR^a^/*wt*^b^ mice, SHM on the *wt*^b^ allele reached 11.565‰ bp (Table 1, Figure 4A and 4B). Interestingly, the Δ3′RR^a^ allele from heterozygous cells reached an intermediate 3.99‰ level, which although below the *wt*^b^ level (*p* < 0.0001) is 4-fold higher than with a biallelic 3′RR defect (*p* < 0.0001) (Table 1, Figure 4A and 4B). Even though the higher error background level with NGS was less adapted for evaluating low-level SHM on the Δ3′RR allele, it confirmed cloning/sequencing data from a previous study [13] and additionally indicated on a high number of reads that SHM hotspots were superimposed in *wt*^b^ and Δ3′RR^a^ alleles in a heterozygous context providing *trans*-complementation (Figure 4C)*, Trans*-complementation by 3′RR^b^ rescues SHM with a typical AID pattern (increasing around RGYW/WRCY sequence targets) although this occurs at a lower level than in *cis*

**Table 1:** Rescued-SHM in Δ3′RR^a^/*wt^b^* mice

	Genotypes	Δ3′RR		Δ3′RR^a^/wt^b^	
	Alleles	Igκ (control)	Allele a (Δ3′RR)	Allele a (Δ3′RR)	Allele b *(wt*)
	Analyzed regions	3′J_κ_5	3′J_H_4	3′J_H_4	3′J_H_4
**B220^+^/GL7^+^**	Total number of mutations	515	663	2034	11,954
	Total length analyzed (base pairs)	30,470	754,252	508,633	1,026,387
	Mutation frequencies (per 1000 base pairs)	16.90	0.89	3.99	11.65
**B220^+^/GL7^+^** **/IgM^+^**	Total number of mutations	143	8	24	135
	Total length analyzed (base pairs)	24,376	49,632	44,462	38,775
	Mutation frequencies (per 1000 base pairs)	5.87	0.16	0.54	3.48

Mutation frequencies in 3′J_H_4 and 3′J_κ_5 introns in B220^+^/GL7^+^ and B220^+^/GL7^+^/IgM^+^ cells from homozygous Δ3′RR and Δ3′RR^a^/*wt*^b^ mice. Data were obtained from at least 2 experiments, ≥2 mice per group, with clonally related sequences counted only once.

Some unsorted VDJ^a^ sequences might have been joined with 3′RR^b^ through *trans*-CSR, then conventionally promoting SHM in *cis*. To exclude that, we also studied sorted unswitched IgM^+^/B220^+^/GL7^+^ cells: as expected SHM was lower but confirmed the above-described variations in 3′RR-less loci compared with either heterozygosity to homozygosity. Δ3′RR homozygous cells showed strongly decreased SHM (0.16‰ for *IgH vs* 5.87‰ for *Igκ*) (Table 1, Figure 4D). By contrast, in Δ3′RR^a^/*wt*^b^ cells, SHM on the Δ3′RR^a^ allele increased by 3-fold (0.54‰) (*p* < 0.01) (Table 1, Figure 4D), although lower than the 3.48‰ yielded in parallel on *wt*^b^ alleles (Table 1, Figure 4D). Accordingly, the 3′RR-deficient allele showed less unmutated sequences when under heterozygosity, but more with 1-3 mutations, while no highly mutated sequence appeared in such IgM^+^ cells (Figure 4D).

Altogether, the 3′RR promotes inter-allelic interactions in both transgenic and endogenous *IgH* loci. In addition to its role in promoting interactions between *IgH* alleles, the 3′RR also supports AID recruitment on S (for CSR) and VH regions (for SHM), not only in *cis* but also in *trans*. We observed allele pairing in *in vitro* stimulated B-cells and measured CSR and SHM in *trans* showing 3′RR-dependent transvection in at least a fraction of activated B-cells. Even if 3′RR-dependent AID-initiated lesions occur less frequently in *trans* than in *cis*, they break the paradigm that the 3′RR is simply a *cis*-regulatory element and demonstrate a missing step that is important for generating the frequent interallelic CSR junctions (found in roughly 20% of B-cells). Functional transvection occurs during the window of time of AID activity and prior to any DNA recombination. Gene *trans*-activation occurs in yeast, drosophila and mammals and *cis-*regulatory elements can interact at long-distance [30]. *IgH* locus physiology is known to involve different types of intra-chromosomal loops that contribute to recombination [19, 31, 32]. In addition, we now demonstrate that the B-cell nucleus also supports *IgH* inter-allelic contacts allowing first transvection and eventually *trans*-CSR. It remains to be determined whether *trans*-interactions reflect bystander proximity after binding identical transcriptional factors, enzymes that may be rate-limiting and only concentrate in a few locations of the nucleus, or whether inter-allelic proximity is functionally important for optimal B cell maturation, by promoting use of the alternate *trans*-CSR pathway.

Non-coding RNAs might contribute to *trans* interactions as they were shown to modulate bridges and potential DNA loops in *cis* between distant *IgH* transcriptional regulatory elements, promoting AID accessibility [4].

Deleterious off-target interactions (which can promote translocations) affect *Ig* loci, and *c-myc* often co-localizes with the *IgH* transcription factory [33, 34]. AID is normally recruited at S regions by 3′RR-dependent chromatin marks [29] but *c-myc* proximity combined with a high frequency of off-target breaks can result in oncogenic translocations [35, 36]. An attractive hypothesis is that transvection between fragile *IgH* loci has evolved beside the major cis-CSR pathway in order to further promote legitimate and safe interactions between homologous alleles. Interestingly, this could then also help out-compete hazardous interactions with illegitimate partners. The 3′RR super-enhancer might thus favor transcription and remodeling of *IgH* loci (together with dangerous partners), and be an intrinsic caretaker of the B-cell genome.

## EXPERIMENTAL PROCEDURES

### Mice

Our research has been approved by the local ethics committee review board. Transgenic 995, 820 and 3´ enhancer deleted 820 (Δ820) mice were a kind gift of Dr. Wesley Dunnick (Figure 1B). 3′RR-mediated inter-allelic *trans*-interactions were studied using a 3′RR-deleted (Δ3′RR) locus (with altered CSR and SHM [16, 17]) either under homozygosity or with heterozygous Δ3′RR^a^ / wild-type (*wt)*^b^ alleles of different *IgH*^a/b^ allotypes (Figure 2A). 3′RR-deficient (Δ3′RR) *IgH*^a/a^ mice were bred with *wt IgH*^b/b^ (C57BL/6) mice or enhancer Eµ-deficient mice (ΔEµ) mice [37] (Figure 2F). 8-10 weeks old littermates were used in all experiments. Allotypes known for several *IgH*^a^ and *IgH*^b^ constant genes can monitor the relative expression of both loci in heterozygous *IgH*^a/b^ mice.

### Heavy chain transgene chromosomal insertion site in lines 995 and 820

Genomic DNA from transgenic lines 995 and 820 was digested with MboI, diluted, and ligated to form circles, some of which include the 3´ end of the transgene and the adjacent chromosomal sequences from the host mouse (either C57BL/6 or SJL). PCR primers were selected for an orientation that would amplify a small portion of the 3´ end of the transgene and the adjacent chromosomal sequences in between. For line 820, the chromosomal sequences matched C57BL/6 chromosome 7 sequences (491 of 497 bases) at position 122 Mb (of 145 Mb total length).

### Cell culture

Splenocytes were collected, red blood cells were lysed and CD43 depleted using CD43 microbeads (Miltenyi Biotec). B splenocytes were cultured 1, 2, 3, 4 or 5 days (for 3D-FISH) or 4 days (for ELISA and ELISpot) in RPMI containing 10% FCS with LPS (20µg/mL) or with anti-CD40 (5µg/ml) (RD systems) + IL4 (40ng/mL) (Peprotech) or LPS (20µg/mL) + IFNγ (2ng/mL) (RD systems).

### 3D-FISH

Interphase DNA FISH was performed as previously described [10] with minor adaptation. In vitro activated B-cells were dropped onto poly-L-lysine slides and fixed with 4% paraformaldehyde for 10 min at room temperature (RT). After washing with PBS, cells were permeabilized with pepsin 0.02% / HCl 0.1M for 15 min at RT, then washed and post-fixed with 1% paraformaldehyde for 5 min at RT, denatured for 5 min in 70% formamide, 2X SSC at 72°C and hybridized overnight at 37°C. The *IgH* (RP23-109B20) probe (encompassing an AID-targeted region from the DH cluster to Cγ2a) and RP23-436M24 was respectively labelled with dCTP-biotin (Invitrogen) or with dUTP-digoxigenin (Roche). Slides were washed in 1X SSC at 72°C for 5 min, incubated with steptavidin-Alexa Fluor 594 (1/200, BD biosciences) or with anti-digoxigenin-Alexa Fluor 488 (Abcam) 1h at RT, and mounted with vectashield containing DAPI (Vector labs). Images were acquired with an epifluorescence microscope (LEICA DMI6000B) or by confocal microscopy on a Leica SP5 AOBS system (Acousto-Optical Beam Splitter). Optical sections separated by 0.2 to 0.3µm were collected and stacks were de-convoluted and analyzed using Huygens and Volocity softwares, respectively. Separation of alleles was measured in 3D from the centre of mass of each signal. Volumetric pixel size was 0.064µm in xy and 0.2 µm in z-direction.

### Chromosome conformation capture (3C)

10^7^ cells were fixed with PBS 10% FCS and 1% formaldehyde for 10 min at RT, stopped with glycine (0.125M). Cells were lysed in Tris 10mM, NaCl 10mM, NP40 2%, supplemented with protease inhibitors, with 10 strokes of a dounce homogeneizer (Pestle A). Nuclei were resuspended in restriction buffer, permeabilized with SDS (0.1%) for 10min at 37°C and triton X-100 (1%) was added. DNA was restricted with HindIII (900U) overnight at 37°C. After HindIII inactivation DNA was ligated with T4 DNA ligase (NEB) overnight at 16°C in 8mL. The crosslink was reversed by proteinase K (500µg) overnight at 65°C and then 300µg of RNAse A was added and incubated 1h at 37°C. DNA was phenol/chloroform extracted and quantified with Qubit (Invitrogen).

The linear range of PCR amplification was determined by serial dilution of the control and the crosslinked chromatin templates. PCR was performed with Herculase Taq polymerase (Agilent) on 200ng DNA using Eµ primer: TTGACATTCTGGTCAAAACGGC and hs4: CAACCTGTGTCCCTAGAGGAGT or calreticulin primers F : CCCAAACCACCACTACCATTACA and R : GATGAACTGCCCTATCCTGAGTC (95°C 2min, 45 cycles 95°C 20sec, 56°C 20sec, 72°C 15sec and 72°C 3min). PCR products were quantified with Image J. Relative crosslinking frequencies were calculated as described [19]. BAC RP23-109B20, 199M11 and RP23-421H21 provided control templates.

### Allotype-specific ELISA

ELISA for the presence of “a” allotype IgA^a^, IgG2a, and IgE^a^ were performed on sera or supernatant from Δ3′RR homozygous, Δ3′RR^a^/*wt*^b^ heterozygous, *wt*^a/b^ and *wt*^b/b^ mice or *in vitro* stimulated B-cells. A pool of *wt*^a/b^ sera was used as standard. Plates were coated with monoclonal antibodies specific for IgA^a^ (clone EC2, BD Pharmingen) or IgG2a (731926, Beckman coulter) overnight, samples were then incubated 2h at 37°C. After washing AP-conjugates goat anti mouse IgA (1040-04, Southern biotech) or IgG2a (1080-04, Southern biotech) were incubated 1h at 37°C. After washing and addition of AP substrate (Sigma), optic density was measured at 400 nm. For ELISA IgE^a^, black 96-well Immuno plates (Thermoscientific) were coated overnight with anti-IgE (732385, Beckman coulter). Samples were incubated 5h at 37°C and, after washing, with biotinylated anti-mouse IgE^a^ (BLE408804, Ozyme) overnight at 37°C. After washing, streptavidin-AP (Sigma) was incubated 45 min at 37°C. After washing, AP activity was assayed using 4-methylumbelliferyl phosphate (Molecular probes) and fluorescence was read at 449 nm.

### ELISpot

For evaluation of IgG2a or IgE^a^ secretion, cells were seeded in duplicate at a density starting at 5 × 10^4^/well, followed by 5-fold serial dilutions in culture medium on a 96-well MultiScreen HTS plate (Millipore) coated with 1.5 µg per well of anti-IgG2a or anti-IgE^a^. Cells were incubated 7 h at 37 °C and then removed by washing with PBS/Tween 0.01 %. Plates were then incubated 1 h at 37 °C with 1 µg/well of alkaline phosphatase-coupled anti-IgG2a or biotinylated-anti-IgE^a^. After washing with PBS/Tween 0.01 %, streptavidin-AP (Sigma) was incubated 45 min at 37°C (for IgE^a^). After washing, addition of 100 µL of BCIP/NBT alkaline phosphatase substrate (Millipore), new washing and drying, images were taken with NI-E microscope (Nikon) and analyzed for spots numbers with the Nis-Ar software (Nikon).

### Flow cytometry and cell sorting

Cell suspensions from Peyer's patches were labeled with anti-B220-APC- (Clone RA3-6B2, Biolegend), GL7-PE- (BD), anti-IgM-FITC (Clone eB121-15F9, ebiosciences) -conjugated antibodies. Sorting of B220^+^/GL7^+^ or B220^+^/GL7^+^/IgM^+^ B-cells was performed on a FACS ARIA 3 (BD Biosciences). Cell sorting quality was validated when SHM frequency at the Igκ locus was superior to 5%.

### Somatic hypermutation analysis

SHM analysis was performed as described [16] from sorted B220^+^/GL7^+^ or B220^+^/GL7^+^/IgM^+^ B-cells cells. For NGS experiment, PCR was performed using primers containing GS junior adaptors and sequences specific of V region (GCGAAGCTTARGCCTGGGRCTTCAGTGAAG) and J_H_4 intron (AGGCTCTGAGATCCCTAGACAG). Amplifications were performed with Phusion^®^ High-Fidelity DNA Polymerase (New England Biolabs) according to the following program: DNA was denatured 30 s at 98°C and then submitted to 42 cycles consisting of 98°C for 10 s, 60°C for 30 s and 72°C for 30 s, and 1 cycle at 72°C for 10 min.

PCR products were first purified using NucleoSpin kit (Macherey-Nagel) and then using Ampure beads (Beckman Coulter). First a PCR emulsion (GS Junior+ emPCR Kit (Lib-A), Roche) was performed and finally PCR products were sequenced using the GS junior sequencing kit XL+ (Roche).

### Statistical analysis

Statistical tests were performed using GraphPad Prism (*p < 0.05, **p < 0.01, ***p < 0.001, ****p < 0.0001).

## SUPPLEMENTARY MATERIALS FIGURES AND REFERENCE



## Abbreviations

IgH: Immunoglobulin heavy chain
CSR: class-switch recombination
3′RR: 3′ regulatory region
AID: activation-induced cytidine deaminase
SHM: somatic hypermutation
