# Supplementary material for: The IgH locus 3′ cis-regulatory super-enhancer co-opts AID for allelic transvection
Source: Oncotarget. 2017 Jan 10;8(8):12929–40. doi: 10.18632/oncotarget.14585 (PMC5355067; doi:10.18632/oncotarget.14585)
Supplement: Supplementary file 1 [file oncotarget-08-12929-s001.pdf]

The *IgH* locus 3’ *cis*-regulatory super-enhancer co-opts AID for allelic transvection

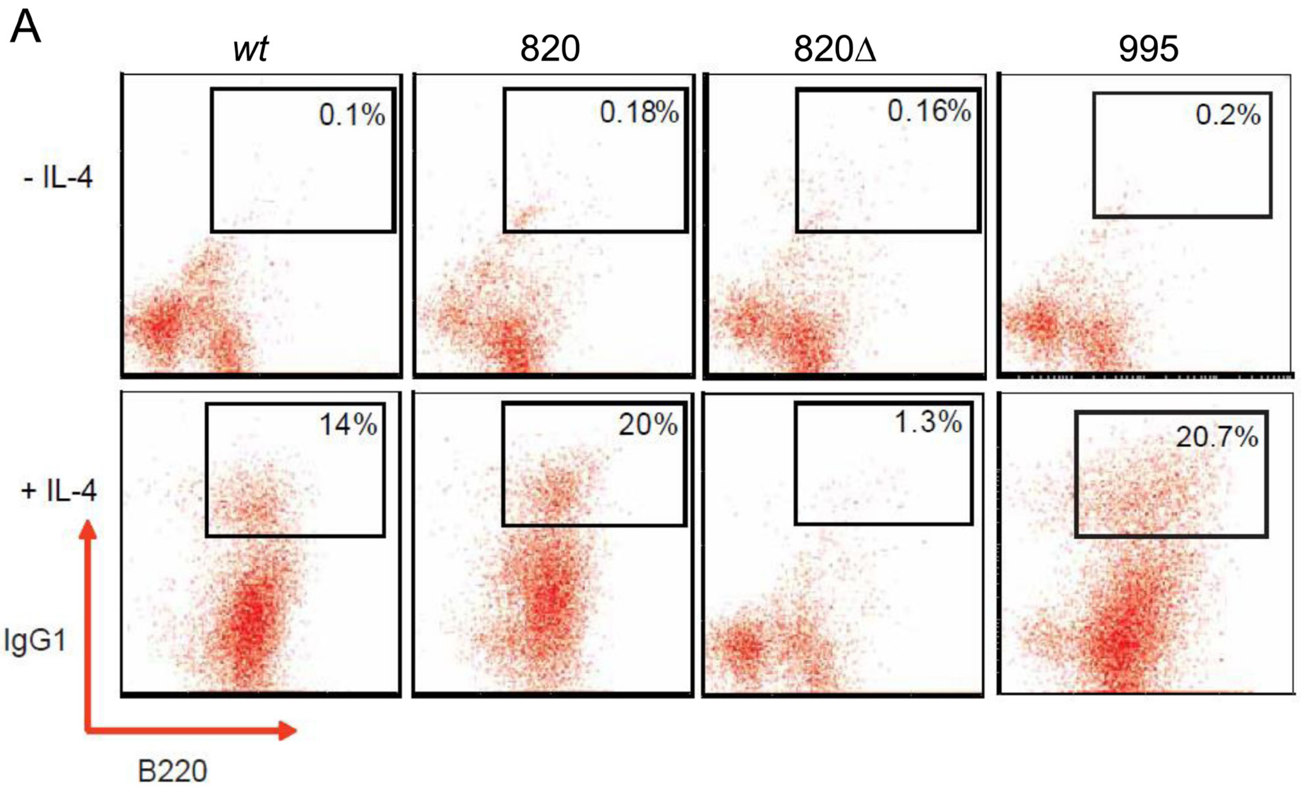

Supplementary Figure S1A: We analyzed mice bearing functionally V(D)J rearranged *Igh* transgenes which inhibit V(D)J rearrangement on both endogenous alleles (Dunnick et al., 2009). When stimulated with LPS + IL4, only the transgenes including a functional 3’RR (820 and 995), but not 820Δ, undergo CSR to IgG1.

B

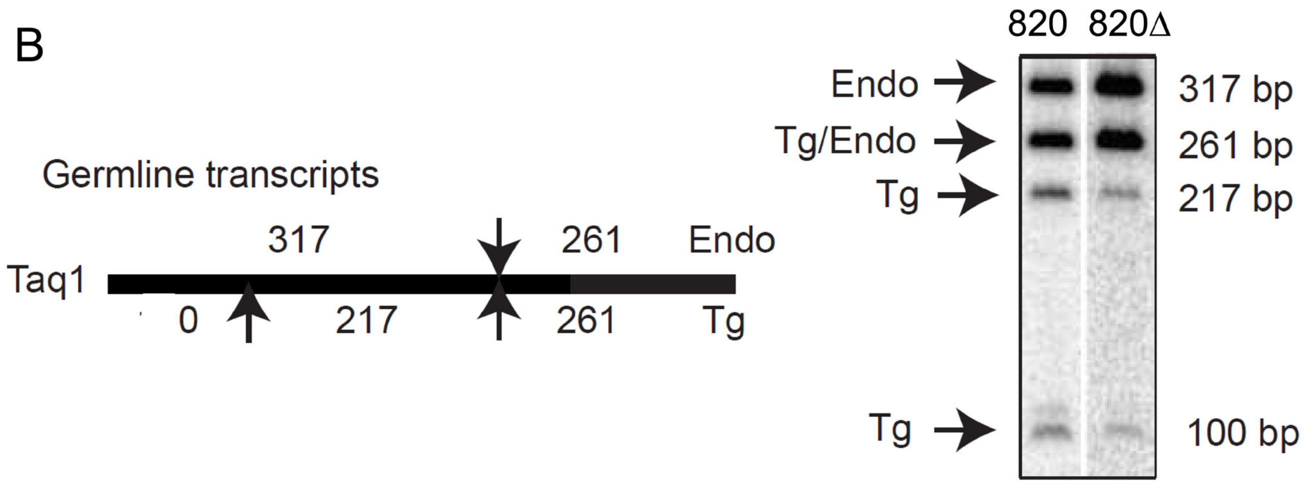

Supplementary Figure S1B: C γ 1 germlines transcripts were studied by RT-PCR as described (Dunnick et al., 2009), in 820 and 820Δ transgenic splenocytes stimulated with LPS+IL-4. Germline transcripts from the endogenous loci and from the transgenes differ for a 4-bp insertion resulting in a TaqI restriction site (arrows).

REFERENCE:

Dunnick WA, Collins JT, Shi J, Westfield G, Fontaine C, Hakimpour P, Papavasilio FN. Switch recombination and somatic hypermutation are controlled by the heavy chain 3' enhancer region. J Exp Med. 206, 2613–2623. doi:10.1084/jem.20091280.
